# Effects of Ageing on the Anterior Segment of the Eye Structure and Function

**DOI:** 10.1155/2018/6260829

**Published:** 2018-05-09

**Authors:** Alejandro Cerviño, Hema Radhakrishnan, José F. Alfonso, Rune Brautaset, José M. González-Meijome

**Affiliations:** ^1^Optometry Research Group, Department of Optics & Optometry & Vision Sciences, Universidad de Valencia, Valencia, Spain; ^2^School of Health Sciences, The University of Manchester, Manchester, UK; ^3^Fernández-Vega Ophthalmology Institute, Oviedo, Spain; ^4^Department of Clinical Neuroscience, Karolinska Institutet, Stockholm, Sweden; ^5^Department of Physics (Optometry), University of Minho, Braga, Portugal

The world will soon have more aged people than children and more people at extreme old age than ever before [1]. Improving quality of life as well as reducing severe disability due to age-related problems has become key for the health systems worldwide. The ageing of the world population has both structural and functional consequences for the human visual system; changes due to ageing occur in all the structures of the eye causing a variety of effects.

Over the last few years, great advances in ophthalmic instrumentation allow the determination of ocular parameters to a level of detail without precedent. Such advances allow researchers to develop specific devices for visual correction and rehabilitation and at the same time guide the clinicians in their decision making and selection of treatment options to convey with the increasing demand of high-quality outcomes of the ageing population.

This special issue aimed at creating a multidisciplinary forum of discussion on recent advances in the knowledge of the effects of ageing in the anterior segment of the human eye's structure and function, and improvements on early detection, treatment, and prognosis of age-related ocular conditions of the anterior segment. As a result, a remarkable compilation of ten articles cover many of these very different aspects of ocular ageing.

Y. Zha et al. report significant biometric differences between preterm infants without retinopathy compared to term infants with interesting implications in refractive development. L. F. Hernandez-Zimbrón et al. carried out an interesting revision of different age-related processes occurring in the different structures of the anterior segment of the eye from a biological perspective and at molecular level. They conclude that the structural and molecular changes observed in the anterior eye segment are caused by molecular changes in intercellular unions, structural arrangements of collagen fibers, overexpression of degradation enzymes, underexpression of inhibitors of metalloproteases in tissues, UV light absorbed, inflammatory cytokines and molecules, and dysregulation of autophagy, among others.

All these changes affect the optical properties of the optical media, and this topic is covered by S. Gholami et al., who investigate the changes in retinal straylight occurring with cataracts of different types and their impact in visual performance, highlighting the importance of including cataract morphology when assessing the visual impact of this age-related condition. M. Jaskulski et al. propose a new refraction metric to better predict spherocylindrical refraction from optical quality metrics for varying pupil sizes. Pupil size determination, on the other side, is suggested by S. Frost and coauthors as an interesting tool for screening preclinical Alzheimer disease since they report changes in pupil flash response in this preclinical phase of the condition.

Pseudoexfoliation syndrome as an age-related complex systemic disorder and its relationship with vascular disease was explored by H. Lesiewska et al., which has been inconclusive from the outcomes of previous reports.

A. K. Schuster et al. report on the distribution of iris conicity using Scheimpflug imaging in a population-based study, part of the outcomes from the Gutenberg Health Study, and test whether pseudophakia allows the iris to sink back. They conclude that steeper conicity was independently associated with a shallow anterior chamber and thicker crystalline lens, while older persons had flatter iris conicity. They also found that cataract surgery flattens the iris position, therefore reducing the risk of angle closure.

The topic of cataract surgery has also been covered by an extensive review by C. Perez-Vives, particularly on current biomaterials used for intraocular lenses and their benefits and complications. Intraocular corrective systems have evolved dramatically over the last decades, providing with advanced designs for overcoming the functional limitations of crystalline lens ageing, that is, loss of transparency and loss of accommodation. But the high incidence of retinal disease also implies a relatively high prevalence of specific aids for visual remains optimization through customized optical-based devices. Those advances allowed the field to evolve into clinical solutions that offer a satisfactory recovery of the capability to see at different distances with customized extended depth of focus and multifocal intraocular lenses. These devices induce, however, some challenges to the patient with the manifestation of dysphotopsia that can now be measured and quantified in the clinical setting. G. Zoulinakis and T. Ferrer-Blasco present a new intraocular telescopic system designed for magnifying retinal image that would benefit eyes with localized retinal damage.

In addition, the advent of technological progress, increasing dependance on mobile technology, and global use of social networks imply that the accommodative system is exposed to new challenges and demands for longer periods of time at any age. R. Montés-Micó et al. explore the accommodative stimulus curve with emoji symbols in an original study that reveals similar responses than those obtained when reading standard text on smartphones.

To summarize, the present special issue accomplished its main aim, that is, novel approaches to age-related functional problems have been proposed, comprehensive reviews on important issues related with ageing of ocular structures and the solutions to the functional problems involved were addressed, and studies on the changes occurring with ageing from different perspectives and from young to old eyes were presented.

*Alejandro Cerviño* *Hema Radhakrishnan* *José F. Alfonso* *Rune Brautaset* *José M. González-Meijome*

## References

[1] World Health Organization (2011).

